# Application of simple swallowing provocation test with fiberoptic endoscopic evaluation of swallowing in a cross-sectional study

**DOI:** 10.1186/s12877-015-0049-5

**Published:** 2015-04-16

**Authors:** Chiharu Tejima, Takeshi Kikutani, Noriaki Takahashi, Fumiyo Tamura, Mitsuyoshi Yoshida

**Affiliations:** Division of Clinical Oral Rehabilitation, the Nippon Dental University, Graduate School of Life Dentistry, Tokyo, Japan; Division of Rehabilitation for Speech and Swallowing Disorders, the Nippon Dental University, Tama Oral Rehabilitation Clinic, Tokyo, Japan; Dental Department, Hiroshima City Rehabilitation Hospital, Hiroshima, Japan

**Keywords:** Swallowing disorders, Dysphagia, Provocation test, Fiberoptic endoscopic evaluation of swallowing (FEES)

## Abstract

**Background:**

This study aimed to develop a simultaneously swallowing provocation test for dysphagia patients undergoing fiberoptic endoscopic evaluation of swallowing (FEES), as well as to evaluate its efficacy.

**Methods:**

In this test, 0·4 and 2·0 mL volumes of water were dripped into the pharynx under endoscopic examination of swallowing, and determine the latency time (LT) of the swallowing reflex elicited by water. The subjects were 51 bed-bound patients with dysphagia and could be divided into two groups as 35 tube feeding and 16 oral intake subjects. Among the tube feeding subjects, 20 patients who started dysphagia rehabilitation were followed-up to 3 months.

**Results:**

The mean LT was 7·43 ± 7·19 seconds with 0·4 mL of test water and 5·05 ± 5·59 sec with 2·0 mL. When 0·4 mL water was dripped, LT was significantly longer in tube feeding (10·49 ± 7·97 seconds) than oral intake subgroup (5·72 ± 5·16 seconds) (p < 0·05). After the dietary intervention according to the result of FEES, 5 patients were improved in eating, and 15 patients were unchanged or even got worse among 3-months follow-up investigation. LT with 0.4 ml of test water of the Improved group was 3·16 ± 2·69 seconds and that of unchanged/worsened group was 22·6 ± 17·5 seconds, resulting in the significant difference (p < 0·05).

**Conclusions:**

The results of this study suggest that our endoscopic swallowing test as swallowing provocation test with FEES is a useful examination for dysphagia rehabilitation.

## Background

Dysphagia is known to increase with age and to be associated with cerebrovascular accident and neuromuscular disease, and to cause malnutrition and aspiration pneumonia [1,2]. Thus, dysphagia is a key to the life prognosis of elderly patients [3]. There are various well-known effective methods of prophylaxis for malnutrition or aspiration pneumonia in dysphagia patients, such as rehabilitation of oropharyngeal function [4], texture-modified diet [5], posture correction, and eating instruction [6], which are performed based on the diagnosis of dysphagia [7,8]. In order to provide appropriate assessment for dysphagia patients, many screening tests to detect dysphagia [9,10] have been applied in clinical settings. In particular, Teramoto et al. [11] reported the Simple Swallowing Provocation Test (SSPT) as a simple and more widely applicable screening test method for dysphagia, focusing on the presence or absence of induction of the swallowing reflex during the pharyngeal phase. This study has advantage to detect silent aspiration especially for unawakened person. Because this SSPT developed for the predicting of pneumonia risk due to saliva aspiration at night, it is unclear whether it can use for the predicting for the possibility of oral feeding.

The fiberoptic endoscopic evaluation of swallowing (FEES) has been proposed, in recent years, as a useful supplementary tool for studying swallowing [12]. During the FEES examination, compensatory positions may be kept to improve swallowing efficacy and therapeutic maneuvers can be performed that can help establish the appropriate rehabilitation approach for managing feeding and swallowing techniques. The FEES is considered to be a good method for establishing the best means of feeding (by mouth, by mouth with dietary restrictions, by tube). Some fibroptic endoscopy has a catheter sheath which can use for water injection, therefore, we have developed an altered swallowing function test accompany with FEES based on the SSPT and evaluated its usefulness in clinical settings.

## Methods

### Participants

The subjects in the patient group were 51 nursing home residents who presented with chief complaint of dysphagia between November 2011 and June 2012 (20 male and 31 female, mean age 83·32 ± 9·08 years). The data included their underlying diseases were extracted from the medical records. The method of their nutrient intake was classified using the Functional Oral Intake Scale (FOIS, Table 1) proposed by Crary et al. [13], as the tube-dependent group (FOIS: 1–3) and total oral intake group (FOIS: 4–7). Prior to the test, we explained the purpose of this study verbally and in writing to the subjects or their caregivers/family members, and obtained their consent. This study was approved by the Ethics Committees for Clinical Research of the Nippon Dental University School of Life Dentistry at Tokyo (Approval number: NDU-T2010-14).

**Table 1:** **FOIS (Functional Oral Intake Scale)**

1	No oral intake
2	Tube dependent with minimal/inconsistent oral intake
3	Tube supplements with consistent oral intake
4	Total oral intake of a single consistency
5	Total oral intake of multiple consistencies requiring special preparation
6	Total oral intake with no special preparation, but must avoid specific food or liquid items
7	Total oral intake with no restrictions

### Procedure

**Figure 1:**
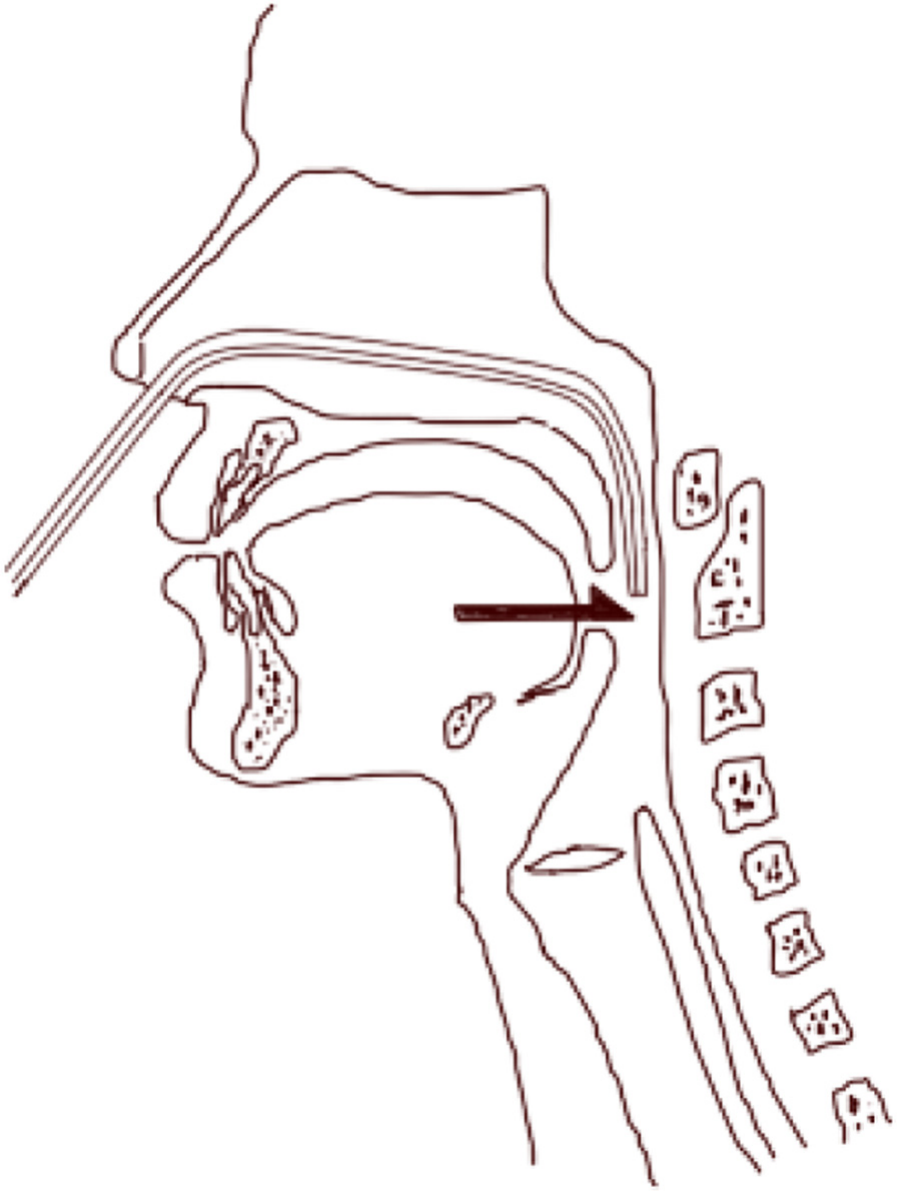
Shema of videoendoscopic swallowing provocation test. The arrow(→) indicated the position of the apex of fibroscope with 5Fr catheter for injection of water.

The details of our endoscopic swallowing test used for this study were as follows (Figure 1). A channeled sheath for prevention of infection (Endoseath®, Medtronic Japan Co., Ltd.) was connected to the endoscopic probe, and a 5Fr catheter for injection of water was attached to the working channel. Then, the endoscopic tube for examination of swallowing was inserted into the nasal cavity according to the manual established (and amended in 2012) by the Medical Review Committee of the Japanese Society of Dysphagia Rehabilitation [14]. The tip of the endoscope with the injection catheter was adjacent to the palatine uvula, injection was started after the subjects were kept on sitting position as same as the normal position for eating. Then, 0·4 mL of colored sterilized physiological saline (25°C) was dripped into the pharynx in one shot using the catheter to evaluate the swallowing dynamics. Dripping was performed at exhalation to evoke smooth swallowing reflex and also prevent aspiration during inhalation [15]. Pharyngeal/laryngeal suctioning was performed in the subjects who had no swallowing or who had aspiration. After confirming that the colored saline in the pharynx/larynx was removed by swallowing or suctioning, 2·0 mL of colored sterilized physiological saline was dripped in the same manner. The injection procedure was repeated three times with each amount of test water and mean value was used for analysis. Latency time (LT) was defined as the period from the start of dripping the test water to the start of swallowing, and was determined from a video shot from the start of dripping of water to the start of swallowing movement. The subjects were observed for 30 seconds after dripping of water, and those without a swallowing reflex were excluded.

Among the tube-dependent group by FOIS, 20 patients who could start dysphagia rehabilitation such as diet modification and posture control were follow-up up to 3 month. LT and the changes of FOIS score were assessed during the follow-up investigation.

### Statistical analysis

Welch’s t- test and chi-square test were used to compare the groups with the aid of SPSS ver.18 for Windows (SPSS Inc. Chicago, IL). A p value <0·05 was considered statistically significant.

## Results

Among the subjects, most common underlying disease was cerebral infarction (46%), followed by dementia (38%) and others (16%). The endoscopic swallowing test revealed that LT was 7·43 ± 7·19 and 5·05 ± 5·59 seconds when 0·4 and 2·0 mL of test water was dripped, respectively.

### Relationship between presence of aspiration detected with FEES and LT

**Figure 2:**
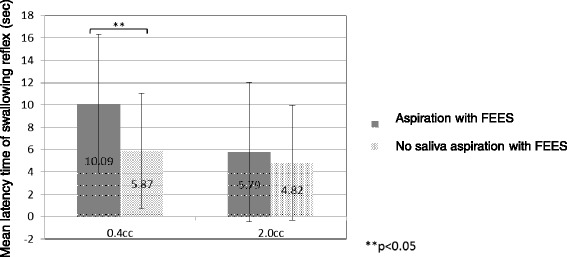
Relationship between presence or absence of aspiration detected with FEES and latency time of swallowing reflex. Error bars represent 1 standard deviation from the mean, the 95% confidence interval. Asterisks indicate difference between the presence of aspiration group (N = 26) and the absence of aspiration group (N = 25) on chi-squared test (p < 0.05).

When 0.4 mL of test water was dripped into the pharynx, LT of dysphagia patients who had saliva aspiration at the time of endoscopy (N = 26) and those without saliva aspiration (N = 25), was 10·09 ± 7·95 and 5·87 ± 5·14 seconds, showing a significant difference between them (p = 0·039). On the other hand, when 2·0 mL of test water was dripped, LT of subjects in the saliva aspiration group and the group without saliva aspiration was not different significantly as 5·79 ± 6·22 and 4·82 ± 5·77, respectively (p = 0·428) (Figure 2).

### Comparison between FOIS and LT

Table 2 shows a comparison between LT and FOIS. LT in the tube-dependent group was significantly longer than total oral intake group as 10·49 ± 7·97 and 5·72 ± 5·16 seconds, respectively (p = 0·011), when 0·4 mL of test water was dripped. On the other hand, LT in the tube-dependent group and total oral intake group was 5·02 ± 5·31 and 4·56 ± 5·72 seconds, respectively, when 2·0 mL of test water was dripped, showing no significant difference (p = 0·994).

**Table 2:** **Comparison between FOIS and LT**

**FOIS**	**N = 51**	**0**·**4 mL (sec)**		**2**·**0 mL (sec)**	
1	31% (16)	10·14 ± 7·36	10·49 ± 7·97	5·8 ± 6·04	5·02 ± 5·31
2	21% (11)	10·77 ± 8·27		7·16 ± 5·72	
3	16% (8)	11·18 ± 10·6		3·6 ± 2·1	
4	16% (8)	6·15 ± 6·03	5·72 ± 5·16	5·99 ± 7·22	4·56 ± 5·72
5	10% (5)	2·24 ± 1·54		2·26 ± 1·05	
6	6% (3)	4·22 ± 3·49		2·87 ± 0·41	
7	0% (0)	―		―	
p-value (Students’ *t*-test)			p = 0·011		p = 0·994

### Relationship between FOIS after 3-month and LT in follow-up patients

There were no patients who had occurred pneumonia during 3-month observation periods. Among the 20 follow-up patient (8 male and 12 female with mean age of 81·2 ± 11·7 years), 5 patients showed improvement in FOIS score after the 3 months. Their LT of 0·.4 ml and 2·0 ml at the first examination were 3·16 ± 2·69 seconds and 3·08 ± 1·33 seconds. The other 15 patients were classified as unchanged/worsened group, and their LT of 0·.4 ml and 2·0 ml were 22·6 ± 17·5 seconds and19·9 ± 22·7 seconds. There was a significant difference between two groups only in 0·.4 ml water test (p = 0.025).

## Discussion

The Simple Swallowing Provocation Test (SSPT), developed by Teramoto et al. [11], is a screening test method for detection of aspiration pneumonia, by dripping 0·4 and 2·0 mL of test water via a nasal tube (inner diameter: 0·5 mm) placed in the oropharynx of subjects in the supine position. It is a simple and more widely applicable screening test method for dysphagia, focusing on the presence or absence of induction of the swallowing reflex during the pharyngeal phase, and its sensitivity and specificity to detect the risk of aspiration pneumonia have been reported to be excellent [16]. However, this test has features that are not suitable for application to the routine assessment of eating/swallowing function by videoendoscopic examination of swallowing in clinical settings, because it requires patients to change their position to the supine position. Therefore, we can challenge developing an altered method for this study, in which an endoscopic probe and a nasal tube are able to be simultaneously inserted using an endoscopic sheath, in order to evaluate both the risk of aspiration pneumonia and possibility of oral feeding. At first, it required us to reconsider the amount of water to be dripped because our study was performed in subjects in a sitting position as same as the normal position for eating. When 0·4 mL of test water was dripped, a significant relationship was observed between LT and the presence of aspiration, while there was no clear relationship when the amount of water was 2·0 mL. The test water was usually deemed to be dripped onto the posterior pharyngeal wall with the method of Teramoto et al. [11], because the subjects were in the supine position, but in our study, test water was dripped into the vallecula of the larynx because the subjects are in a sitting position. It may be one reason why our LT was prolonged than the previous studies [17]. In addition, dripping of 2·0 mL of test water into the vallecula of the larynx was observed to be distressing in some patients with poor pharyngeal sensation and to induced the aspiration before swallowing. Therefore, the appropriate amount of test water in this study was considered to be 0·4 mL. Moreover SSPT detect the swallowing reflex on inspection, which means the start of laryngeal elevation. On the other hand our study determined the swallowing reflex as the white-out of endoscopic viewing, which means the top of laryngeal elevation. It may be another reason why our LT was prolonged than the previous studies. When 0·4 mL of test water was used, LT was significantly longer with tube feeding patients and patients than oral intake patients. It may indicate that the swallowing reflex quality may have an important role in the oral intake ability. It is emphasized with our follow-up study that significant relationships between improved FOIS score and LT for evoked swallowing reflex. There are three types of aspiration, including “aspiration before swallowing” which is defined as aspiration before induction of the swallowing reflex or before closing the larynx, “aspiration during swallowing” which occurs due to insufficient laryngeal closure during the period from the start to the end of the swallowing reflex, and “aspiration after swallowing” in which pharyngeal residue falls into the airway after the end of the swallowing reflex [18]. SSPT was used as an index to estimate whether the speed of the swallowing reflex, considering the timing of swallowing in such patients with poor pharyngeal sensation. Therefore, this test method could provide useful information on the risk of aspiration after swallowing. However, whether the SSPT detects aspiration or penetration before or during swallow correctly is unclear. Recently, Kagaya et al. [19] determined the sensitivity, specificity, and predictive accuracy of SSPT followed by videofluoroscopic examination of swallowing (VF) and concluded that SSPT has limited applicability as a screening tool for aspiration, silent aspiration, or penetration because of its low sensitivity. It may be the reason that SSPT is designed to detect the risk of saliva aspiration at night however VF as well as FEES is to detect the risk of oral feeding at daytime. Therefore, the most advantage of our developed method was that endoscopic imaging and the concurrent simple endoscopic swallowing test enabled us to observe aspiration and choking on the test water at the same time, which might contribute to prevention of aspiration pneumonia and rehabilitation for oral feeding in dysphagia clinics.

## Conclusion

With the limited conditions of this study, it may conclude that our developed endoscopic swallowing test is useful both detect the risk of saliva aspiration at night and the possibility of oral feeding. FEES is now a first choice method for studying swallowing disorders on account of the various advantages it offers: easy to use, very well tolerated, allows bedside examination and is economic [20]. Our developing method will promote this clinical practice.

## Abbreviations

FEES: Fiberoptic endoscopic evaluation of swallowing
LT: The latency time
FOIS: The Functional Oral Intake Scale
SSPT: The Simple Swallowing Provocation Test
VF: Videofluoroscopic examination of swallowing
